# Masked Morphological Priming in German-Speaking Adults and Children: Evidence from Response Time Distributions

**DOI:** 10.3389/fpsyg.2016.00929

**Published:** 2016-06-21

**Authors:** Jana Hasenäcker, Elisabeth Beyersmann, Sascha Schroeder

**Affiliations:** ^1^MPRG Reading Education and Development, Max Planck Institute for Human DevelopmentBerlin, Germany; ^2^Laboratoire de Psychologie Cognitive, Aix-Marseille Université and Centre de la Recherche ScientifiqueMarseille, France

**Keywords:** morphological processing, reading development, masked priming, distributional analysis, visual word recognition

## Abstract

In this study, we looked at masked morphological priming effects in German children and adults beyond mean response times by taking into account response time distributions. We conducted an experiment comparing suffixed word primes (*kleidchen-KLEID*), suffixed nonword primes (*kleidtum-KLEID*), nonsuffixed nonword primes (*kleidekt-KLEID*), and unrelated controls (*träumerei-KLEID*). The pattern of priming in adults showed facilitation from suffixed words, suffixed nonwords, and nonsuffixed nonwords relative to unrelated controls, and from both suffixed conditions relative to nonsuffixed nonwords, thus providing evidence for morpho-orthographic and embedded stem priming. Children also showed facilitation from real suffixed words, suffixed nonwords, and nonsuffixed nonwords compared to unrelated words, but no difference between the suffixed and nonsuffixed conditions, thus suggesting that German elementary school children do not make use of morpho-orthographic segmentation. Interestingly, for all priming effects, a shift of the response time distribution was observed. Consequences for theories of morphological processing are discussed.

## Introduction

In recent years, much research has investigated the role of morphemes in word recognition. Particularly, the mechanisms and time-course of morphological decomposition have been given much attention. One widely used method to examine morphological processing in adults and children is the masked priming paradigm, in which a morphologically related or a pseudo-morphological prime is presented very shortly before the target. Findings from those studies have given rise to the distinction between early automatic processes based on orthography, therefore called morpho-orthographic decomposition, and subsequent processes based on semantic relationships, called morpho-semantic decomposition (e.g., Rastle et al., 2004). Although this distinction is disputed (e.g., Giraudo and Grainger, 2001; Giraudo and Voga, 2014; Feldman et al., 2015), skilled readers have repeatedly been shown to exploit morphology in word recognition by using highly automatized rapid morpho-orthographic decomposition (for a review see Rastle and Davis, 2008). Evidence on the mechanisms underlying morphological processing in children has been mixed (Casalis et al., 2009; Quémart et al., 2011; Beyersmann et al., 2012). Whether children's use of morphemes in visual word recognition is similar to those of adults therefore remains a matter of debate. Crucially, previous masked priming studies have only focused on mean response time differences (but see Andrews and Lo, 2013). This might conceal differences that only arise in a certain portion of the response time distribution: priming effects might occur to different degrees for shorter and longer response times. If priming is modulated by the time processing takes to unfold, this would indicate that it is not a general automatic mechanism. Contrasting the response time distributions of truly morphologically related prime-target pairs and pseudo-morphological pairs therefore promises a possibility to distinguish whether the underlying decomposition mechanisms are the same. Moreover, comparing the response time distributions of adults and children could yield new insights as differences would indicate that the underlying processing mechanisms differ between the groups.

Theories of morphological processing vary considerably in their assumptions concerning the underlying mechanisms. Some claim that all known words are, at least initially, retrieved as full forms (e.g., Butterworth, 1983; Giraudo and Grainger, 2001), others state that sublexical decomposition is obligatory (Taft and Forster, 1975; Taft, 2003) and think of it in terms of *affix-stripping* that acts on any word that *appears* to have a morphological structure. Form-then-meaning accounts (e.g., Rastle and Davis, 2008) and hybrid models (e.g., Diependaele et al., 2009) depict an early sublexical (morpho-orthographic) processing stage, followed by a later meaning-based (morpho-semantic) processing stage (see also Giraudo and Voga, 2014, proposing a sublexical level that is not morphological in nature, but captures the surface structure of affixes, termed *morphomes*). Form-and-meaning accounts (e.g., Feldman et al., 2015), however, assume involvement of semantics already at the earliest stages of word recognition, rendering the morpho-orthographic/morpho-semantic distinction obsolete, as do models such as the amorphous model (Baayen et al., 2011) or the triangle model (Seidenberg and Gonnerman, 2000), that see morphology not as distinct processing units, but as emerging entirely from form-meaning overlap. The different views are often tested by using masked priming experiments.

In the masked priming paradigm, words are preceded by the relatively short presentation (~50 ms) of a related suffixed word (*teacher-TEACH*), a pseudosuffixed word (*corner-CORN*, where *corner* is not the real suffixed derivate of the stem *corn*), or a non-suffixed control (*turnip-TURN*, where –*ip* is not a suffix combining with the stem *turn*; see Rastle et al., 2004). The general findings from several languages (e.g., Dutch: Diependaele et al., 2005; English: Rastle et al., 2004; French: Longtin and Meunier, 2005; Hebrew: Frost et al., 1997; Spanish and Basque: Duñabeitia et al., 2007; see also Rastle and Davis, 2008, for a review) are that stem target recognition is facilitated when preceded by any suffixed prime, regardless of whether it is truly suffixed or pseudosuffixed, relative to any non-suffixed prime. A variation of the masked morphological priming paradigm was introduced by Longtin and Meunier (2005) who used morphologically complex nonword primes that were either interpretable (*rapidifier-RAPIDE*) or non-intertpretable (*sportation-SPORT*) in comparison to real suffixed word primes (*rapidement-RAPIDE, sportif-SPORT*). They found priming from complex nonword primes, independent of the interpretability. From nonwords with nonmorphological endings (*rapiduit-RAPIDE*) they found no priming effects. Using nonwords as primes has several advantages. A first benefit of the nonword paradigm over the word paradigm is the option to pair different prime types with the same targets, which is intricate and very restricted with words (but see Giraudo and Grainger, 2001; Feldman et al., 2015). Moreover, it circumvents the classification into truly suffixed versus pseudosuffixed words, which is problematic as this is often a continuum rather than two distinct categories (see also Beyersmann et al., 2015a). Third, no lexical competition or inhibitory effects can arise from the nonword primes: in a pair like *rapiduit-rapide, rapiduit* should not interfere with *rapide*, while in a *turnip-turn* pair *turnip* might interfere with *turn* (Beyersmann et al., 2015a). Even if a semantic interpretation for a nonword prime is created “on fly” it would necessarily be related to the stem and thus exert a facilitative, but not an inhibitory effect if having an effect from semantics at all. This is important, because it also affects the predicted pattern of priming: when using nonword primes, priming from the stem can be observed also with a non-suffix ending, because facilitation from the stem is not countered by inhibition from the whole word. In a recent study, Beyersmann et al. (2015a) made use of the nonword paradigm by carrying out a masked primed lexical decision study in which the same target (TRISTE) was primes by a suffixed word (*tristesse*), a suffixed nonword (*tristerie*), and a nonsuffixed nonword (*tristald*) in comparison to unrelated words. The results revealed that participants with higher levels of language proficiency showed equal magnitudes of priming across all three conditions, whereas individuals with comparatively lower levels of language proficiency showed significantly more priming in the two suffixed conditions relative to the non-suffixed condition. While the results in the low-proficiency group replicate the findings reported by Longtin and Meunier (2005), the pattern seen in high-proficiency participants suggests that these individuals benefit from the activation of embedded stems, independently of whether they occur in combination with an affix or a non-morphemic ending (for converging evidence, see also Morris et al., 2011; Beyersmann et al., 2016). These results thus suggest that the visual recognition of morphologically complex letter strings is not uniquely based on morpho-orthographic segmentation mechanisms, but that these are at least complemented to some extent by the activation of embedded stems. Taken together, masked morphological priming studies yield effects indicative of early and automatic decomposition that is independent of a pre-existing semantic relationship between prime and target. The nonword paradigm additionally provides new evidence on the priming of stems as an additional mechanism in masked morphological priming.

Another important issue concerning masked morphological priming, that has gained increasing attention in the recent years, is when and how the observed effects emerge in the course of reading development and how this fits with the different models of morphological processing. However, evidence from masked priming in children is still rather sparse and inconclusive, despite the fact that morphology is known to be of great importance in reading acquisition, particularly in languages that are morphologically productive and have a shallow orthography, such as Finnish, Italian or German. Due to their prominence and high reoccurrence, morphemes appear to be sensible devices to make use of in reading. Especially developing readers benefit from breaking down complex words into smaller parts. Previous studies on morphology in language development have supplied evidence that children use morphological knowledge to learn new complex words (Bertram et al., 2000), as well as to spell words (Deacon and Bryant, 2006). Beyond helping accessing the meaning and spelling of a complex word, morphological structure can also be exploited to recognize written complex words efficiently (Carlisle and Stone, 2005). Therefore, investigation of morphological decomposition in children is interesting not least because it allows drawing inferences important for accounts of reading development.

An initial morphological priming study with children, conducted by Casalis et al. (2009), looked at facilitation from morphologically related primes (*laveur-LAVAGE*) and orthographic primes (*lavande-LAVAGE*) in comparison to unrelated primes and found equal effects of morphological and orthographic priming, thus not indicating morphological, but rather orthographic priming when primes were masked (but morphological priming in an unmasked experiment). However, no pseudosuffixed primes were included. Therefore, it is not possible to further distinguish between morpho-orthographic and morpho-semantic priming mechanisms. Pseudosuffixed priming was examined in a related study with French third, fifth, and seventh graders by Quémart et al. (2011), who observed equal priming from both real suffixed and pseudosuffixed primes, but not from nonsuffixed, orthographic primes for children of all grades. The authors propose that children use morpho-orthographic decomposition. These findings are contrasted by evidence from English-speaking children (Beyersmann et al., 2012), showing priming effects only for real suffixed primes, but not for pseudosuffixed or nonsuffixed primes. The authors conclude that priming only arises for semantically related prime-target pairs and morpho-orthographic decomposition is not yet automatized in children. A recent study by Beyersmann et al. (2015b) and the first using suffixed and nonsuffixed nonword primes with children suggests that priming is modulated by reading proficiency: morpho-semantic priming from suffixed words was evident in children across all grades in elementary school, but more proficient child readers additionally showed effects of embedded stem priming from suffixed and nonsuffixed nonwords. As in Beyersmann et al.'s (2012) earlier findings, there was no evidence for morpho-orthographic processing in primary school children.

Crucially, conclusions about the presence or absence of certain priming effects in both adults and children are usually based on differences in mean of response times to conditions. As Balota et al. (2008) point out, relying on differences in means when comparing conditions assumes similar underlying distributions of RTs and a mere shift of the entire distribution. This underscores the likely possibility that RT distributions are differentially skewed. A certain priming condition cannot only shift the whole distribution relative to another condition, but can also affect a certain portion of the distribution. For example, a priming effect can be especially pronounced in longer response times, thus leading to a skew of the distribution. Distributional analyses thus present a promising tool to capture differences in priming effects that might be covered or blurred when using the standard practice of comparing mean RTs. One method to determine differential influences on the RT distribution is by using so-called Vincentiles (Vincent, 1912) or Quantiles. For vincentile or quantile analyses, raw RTs for each participant in a certain condition are ordered from fastest to slowest and grouped into bins (i.e., first 10%, second 10%, etc.). Vincentiles are especially useful to visualize the distribution of RTs in a certain condition: each vincentile can be collapsed across participants and then be plotted. Also, differences between conditions, for example suffixed word primes and unrelated primes, across vincentiles can be plotted to illustrate how the priming effect changes from shorter to longer RTs. Furthermore, they can be used as an informative factor in inferential testing for significance to find out whether short and long reaction times are affected differently by certain primes. The priming effect can remain constant or decrease/increase across vincentiles, thus mirroring a differential impact on certain portions of the distribution. Thus, this technique might provide an informative exploratory extension to the traditional comparison of means.

The vincentile or quantile approach has already provided valuable insights into various processes and limitations of semantic priming (i.e., Balota et al., 2008; de Wit and Kinoshita, 2015). In the context of masked morphological priming, to our knowledge, it has only been applied once so far. Andrews and Lo (2013) used quantiles to investigate individual differences of masked morphological priming with the word paradigm in adult readers. They compared the RT distributions of priming effects in participants with an “orthographic profile” (i.e., relatively better spelling than vocabulary skills) to those of participants with a “semantic profile” (i.e., relatively better vocabulary than spelling skills). Overall, the authors report a significant distributional shift in the RT distribution for transparent (*teacher-TEACH*) and opaque (*archer-ARCH*) pairs relative unrelated pairs and a significantly smaller shift for form pairs (*brothel-BROTH*). The authors discuss this in terms of a headstart activation from primes to relevant targets. Furthermore, the distributional effects were moderated by the participants' profile. In particular, while all participants showed an increase in priming from transparent pairs across the RT distribution, participants with a semantic profile showed decreased priming from opaque pairs in the slower quantiles, and participants with an orthographic profile showed a slight decrease from form pairs also in the later quantiles. The results by Andrews and Lo (2013) clearly demonstrate that the distributional approach is a promising tool for the exploration of masked morphological priming in different participant samples.

To investigate morphological priming in German adults and children with the nonword paradigm, we carried out a masked priming study using real suffixed words (*kleidchen-KLEID*, “little dress-DRESS,” analogous to Eng. *farmer-FARM*), suffixed nonwords (*kleidtum-KLEID*, analogous to Eng. *farmation-FARM*), nonsuffixed nonwords (*kleidekt-KLEID*, analogous to Eng. *farmald-FARM*), and unrelated controls (*träumerei-KLEID*, analogous to Eng. *dreamer-FARM*) as primes. To our knowledge, we are the first to explore suffixed nonword priming in German-speaking individuals. For adults, we expect increased priming in the two suffixed conditions relative to the control condition, in line with the typical findings from previous studies in other languages (Frost et al., 1997; Rastle et al., 2004; Diependaele et al., 2005; Longtin and Meunier, 2005; Duñabeitia et al., 2007; Rastle and Davis, 2008), indicating that the morphemes of the prime are activated in separation, regardless of the lexicality of the prime, thus facilitating target recognition. Moreover, considering recent nonword priming studies (Morris et al., 2011; Beyersmann et al., 2015a, 2016), embedded stem priming indicated by facilitation from nonsuffixed nonwords is also expectable.

For children, the case is less clear-cut. If it is true that young children use morpho-orthographic decomposition as evidence for word primes by Quémart et al. (2011) suggest, we would expect priming in both suffixed conditions but not in the non-suffixed condition. However, if German children do not automatically segment all affixed stimuli, priming should only occur from truly suffixed word primes, replicating the Beyersmann et al. (2012) pattern. Importantly, considering that we use nonword primes, which increases the chances for embedded stem priming effects to arise because no conflicting inhibition can occur as in *turnip-turn* pairs, child readers might also show priming in the nonsuffixed nonword condition (Beyersmann et al., 2015b), if they are able to extract stems.

For the exploratory investigation of the RT distributions it is of special interest: (1) whether priming shifts and/or skews the RT distribution, (2) whether the RT distribution is affected differently in the different priming condition. A shift is usually interpreted as reflecting early pre-activation leading to a head start on target processing, while a skew only affecting the longer response times is indicative of a later process such as feedback activation or evidence accumulation (Balota et al., 2008; Yap et al., 2008; de Wit and Kinoshita, 2015). In this way, certain patterns of response time distributions can be associated with certain accounts of morphological decomposition. Early automatic pre-activation of the target from all suffixed primes, as indicated by a *shift* in the RT distribution in the two suffixed conditions, as Andrews and Lo (2013) found for transparent and opaque pairs when averaging across all participants, is compatible with obligatory decomposition accounts (e.g., Taft, 2003), form-then-meaning accounts (e.g., Rastle and Davis, 2008) and hybrid models (e.g., Diependaele et al., 2009) due to a headstart from morpho-orthographic segmentation. Form-then-meaning accounts additionally suppose later activation from truly-suffixed words due to feedback from morpho-semantic analysis, as do supralexical accounts (e.g., Giraudo and Grainger, 2001), which could manifest in a *skew* of the RT distribution in the suffixed word condition. Finally, a shift in all three related conditions would speak in favor of the early activation of the embedded target word, independently of whether it appears with an affix (*kleid* + *tum*) or a non-morphemic ending (*kleid* + *ekt*). Including quantiles in the analysis thus allows to compare the underlying processes of morphological decomposition and learn about the distinctiveness between early, orthography-based and later semantic-based processing as hypothesized by the different accounts. Considering the patterns for adults and children in conjunction can also shed light on possible differences in the nature of morphological decomposition between skilled and developing readers.

## Method

### Participants

Twenty-four university students (13 women, *M*_age_ = 25.2 years, age range: 20–29 years) from the Berlin area participated for monetary reimbursement. Moreover, forty children (20 girls, *M*_age_ = 8.58 years, age range: 7–10 years, grade 2–5) took part in the study for a small compensation. All participants reported to be native speakers of German. The study took place at the test center of the Max Planck Institute for Human Development (MPIB), Berlin. It was carried out with approval of the MPIB Ethics Committee. All adult subjects gave written informed consent in accordance with the Declaration of Helsinki. For the child participants, written consent was obtained from the parents and oral consent was asked from the children.

In order to test whether the adults and children in our study were representative readers of their age group, we used the 1-min-reading test for words and nonwords from the SLRT-II (Moll and Landerl, 2010). Mean percentiles were slightly above the norm for both groups for words (adults: *M*_*Perc*_ = 68.20, *SD*_*Perc*_ = 20.28, children: *M*_*Perc*_ = 57.96, *SD*_*Perc*_ = 25.67) as well as nonwords (adults: *M*_*Perc*_ = 71.55, *SD*_*Perc*_ = 21.87, children: *M*_*Perc*_ = 53.30, *SD*_*Perc*_ = 28.92).

### Materials

Fifty words were selected as targets. In order to make the experiment also suitable for children, the words were chosen from the childLex corpus (Schroeder et al., 2015). For each target word, four prime conditions were chosen: a suffixed word prime (*kleidchen-KLEID*), a suffixed nonword prime (*kleidtum-KLEID*), a nonsuffixed nonword prime (*kleidekt-KLEID*), and an unrelated prime (*träumerei-KLEID*). Suffixed word primes were existing suffixed forms of the target words (an English equivalent could be *farmer-FARM*). Suffixed nonword primes were created by combining the target words with a different suffix, thus creating a non-existing derived form (an English equivalent being *farmation-FARM*). Nonsuffixed nonword primes were a combination of the target words with non-morphemic endings (equivalent to English *farmald-FARM*). Unrelated primes were existing suffixed words with all letters different from the target word. In total then, half of the critical prime conditions were words and half were nonwords and three of the four conditions shared a stem with the target (see Table A1). All prime conditions were matched on length. Each suffix or non-morphemic ending occurred in 5 different contexts per condition (e.g., *kleidchen, stückchen, pferdchen, steinchen, spielchen*). In total, 10 different suffixes and 10 different non-morphemic endings were used, because existing and non-existing combinations used the same suffixes with different stems. Half of the suffixes were of high normalized type frequency (*-ung, -lich, -ig, -nis, -heit*: *M* = 1281) and the other half of low normalized type frequency (*-chen, -tum, -lein, -ei, -los: M* = 173). Likewise, half of the non-morphemic endings were of high type frequency (*-ucht, -men, -atz, -pfen, -am: M* = 599) and half of low type frequency (*-au, -ekt, -pern, -nauf, -arf: M* = 141). High and low frequency primes were matched on length, suffix length and non-morphemic ending length across conditions.

Fifty nonword targets were created by selecting 50 words from the childLex corpus (Schroeder et al., 2015) and replacing one letter in each word. Primes for nonwords were created following the same principles as for the word targets with the same suffixes and non-morphemic endings. Nonword and word targets and primes were matched on length.

In order to make the stimulus set dividable by four, six filler target words and six filler target nonwords with their respective primes were added, resulting in a total of 112 targets with four possible primes each. From that, four counterbalanced lists were created, each containing a target word only once, such that participants saw each target only in one of the four prime conditions. Per condition, each participant thus saw 12 items.

### Procedure

Participants were tested individually in a quiet room. The experiment was run on a 15″ laptop monitor with a refresh rate of 60 Hz. Stimuli were presented in white 20-point Courier New font in the center of a black screen. Each trial started with a 500-ms forward mask of hash marks followed by a prime in lowercase for 50 ms, directly followed by the target in uppercase. The target remained on the screen until a response was made by the participant. Participants were instructed to decide as quickly and as accurately as possible whether the presented stimuli was an existing German word or not and indicate this by pressing the *D* or the *K* key on a standard keyboard. They were not informed about the prime.

## Results

As usually observed for the lexical decision task in a transparent orthography like German, overall response accuracy was rather high for adults (*M* = 97.2 %, *SD* = 16.6%) as for children (*M* = 91.6%, *SD* = 27.8%). As a consequence, analyses focused on response times. Moreover, main analyses focused on word targets. Incorrect responses were removed, as were response times below 300 ms or above 6000 ms (adults: 0%, children: 1.3%). Response times were then logarithmically transformed and further outliers were trimmed for adults and children separately using model criticism based on a simple model including random slopes for subject and item (Baayen and Milin, 2010) and excluding all data points with residuals exceeding three standard deviations (adults: 1.5%, children: 1.1%). Descriptive statistics for the four priming conditions are provided in Table 1 for adults and children, respectively.

**Table 1:** **Mean response times (in ms) per prime type for Adults and Children**.

	**Prime Type**			
	**Suffixed word**	**Suffixed nonword**	**Nonsuffixed nonword**	**Unrelated word**
Adults	599^a^	602^a^	618^b^	634^c^
Children	1280^a^	1293^a^	1297^a^	1333^b^

*Means with different indexes are significantly different at p < 0.05*.

Data analyses were performed for adults and children separately using (generalized) linear mixed-effects models (Baayen et al., 2008) as implemented in the lme4 package (Version 1.1-6; Bates et al., 2014) in the statistical software R. Prime Type (suffixed word vs. suffixed nonword vs. nonsuffixed nonword vs. unrelated word) was entered into the models as a fixed effect. In order to take into consideration possible differences in the response time distributions, Quantile was also included as a fixed effect. Quantiles were computed by sorting the response times from the shortest to the largest into four bins for each participant and priming condition. Suffix Frequency (high vs. low) was entered to control for potential effects due to differential frequencies (see Beyersmann et al., 2015a). However, it did not improve the models' fit and was therefore dropped from the analyses. Random intercepts were included for participants and items. Model details are shown in Table 2.

**Table 2:** **Results from Mixed-Effects models with prime type and Quantile as fixed effects, and participant and word as random Intercepts**.

	**Adults**			**Children**		
	**χ^2^**	***df***	***p***	**χ^2^**	***df***	***p***
Intercept	73206.00	1	< 0.001	12775.33	1	< 0.001
Prime type	113.98	3	< 0.001	16.26	3	< 0.001
Quantile	1070.09	9	< 0.001	1519.97	9	< 0.001
Prime type × Quantile	15.36	27	0.964	12.96	27	0.990

*Model evaluation using Type III sum of squares and Wald χ^2^ tests with Kenward-Roger df*.

The response time analysis for adults showed a significant main effect of Prime Type, suggesting differential priming effects in the different conditions. Moreover, a main effect of Quantile was present, which was not moderated by Prime Type, indicating that the RT distributions were equally affected in the different conditions. *Post-hoc* contrasts investigating the main effect of Prime Type were calculated with the multcomp package (Version 1.3-3; Hothorn et al., 2008). They revealed significantly faster responses in the suffixed word and suffixed nonword condition compared to the unrelated condition, *z* = 9.43, *z* = 8.60, both *p* < 0.05. Responses were also faster in the nonsuffixed nonword condition compared to the unrelated condition, *z* = 4.15, *p* < 0.05. Moreover, responses in the suffixed word and suffixed nonword condition differed significantly from the nonsuffixed nonword condition, *z* = 5.31, *z* = 4.50, both *p* < 0.05, while there was no difference between the two suffixed conditions, *z* < 1, *p* > 0.05. This pattern indicates that both suffixed words and suffixed nonwords are morphologically decomposed in adult readers of German.

**Figure 1:**
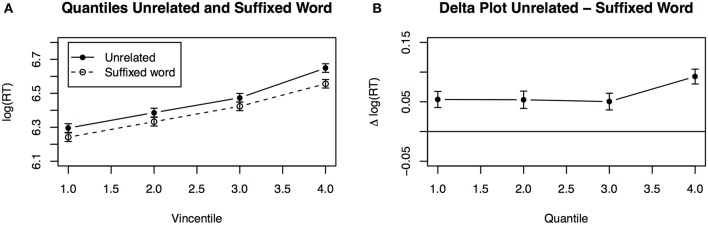
**(A)** Mean values for the unrelated and the suffixed word condition for adults. **(B)** Difference between the unrelated and the suffixed word condition for adults (delta plot).

**Figure 2:**
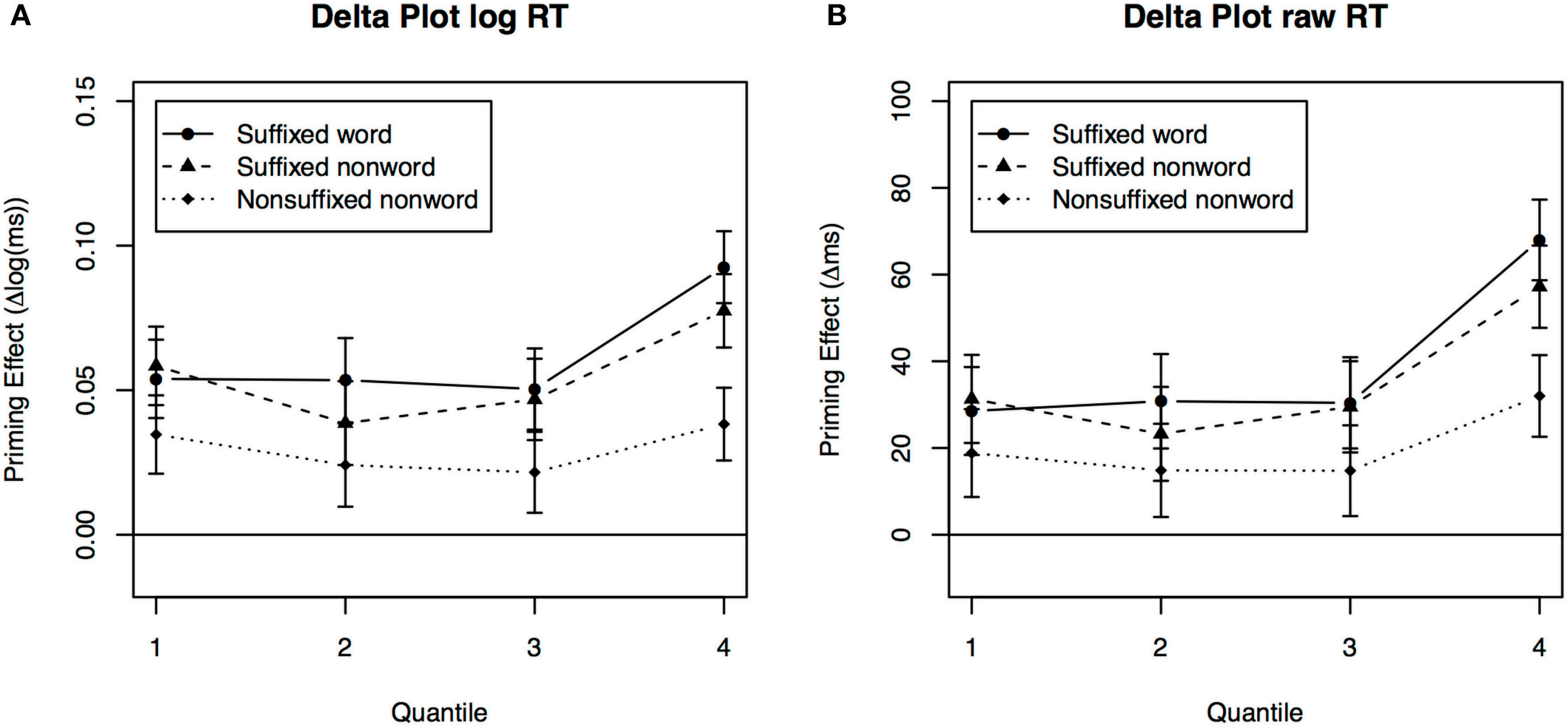
**Delta plots between conditions for adults**. Priming effect of each condition relative to the unrelated condition for each quantile using **(A)** logarithmically transformed RTs and **(B)** raw RTs.

In order to explore the main effect of Quantile in more detail, delta plots were used. Delta plots show the difference between two priming conditions directly. For example, Figure 1A shows the mean response times across quantiles averaged over participants for suffixed words and unrelated words. As one can see, the RTs increase across quantiles in a parallel fashion for both conditions. A delta plot, as in Figure 1B, is created from this by substracting the suffixed from the unrelated condition. The delta plot thus illustrates the priming effect of suffixed relative to unrelated words, which remains constantly above zero across quantiles. This pattern indicates a distributional shift, rather than a skew. Figure 2 illustrates a shift for suffixed nonwords and nonsuffixed nonwords relative to unrelated words.

**Figure 3:**
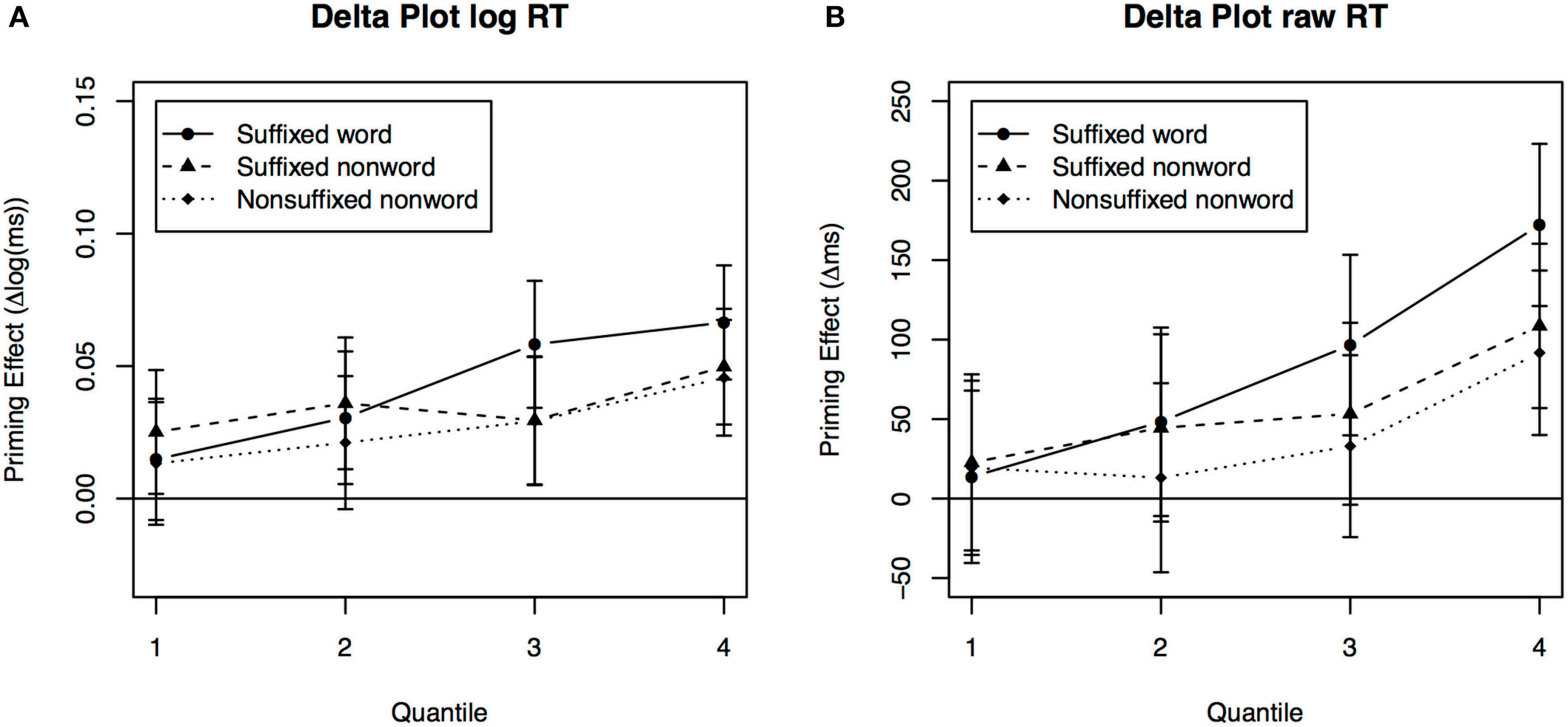
**Delta plots between conditions for children**. Priming effect of each condition relative to the unrelated condition for each quantile using **(A)** logarithmically transformed RTs and **(B)** raw RTs.

The linear mixed-effects model for the children's response times showed a significant effect for Prime Type and a significant effect for Quantile, but no interaction. *Post-hoc* contrasts showed significantly faster responses following suffixed word primes compared to the unrelated condition, *z* = 3.87, *p* < 0.05. Responses in the suffixed nonword and nonsuffixed nonword condition were also faster compared to the unrelated condition, *z* = 2.87, *z* = 2.57, both *p* < 0.05. However, responses in the suffixed word and suffixed nonword condition did not differ significantly from the nonsuffixed nonword condition, both *z* = 1.27, *p* > 0.05, neither did the two suffixed conditions differ from each other, *z* < 1, *p* > 0.05. This pattern suggests that children show facilitation from primes sharing the stem with the target, also in the absence of a suffix. To investigate quantiles for children, we again used delta plots as shown in Figure 3. Although delta plots for children are more noisy, the pattern overall indicates a moderate distributional shift for all related primes (suffixed word, suffixed nonword, and nonsuffixed nonword) relative to unrelated primes.

We also ran similar analyses for the nonword targets. However, as expected, we did not find a significant effect of PrimeType, neither for adults (χ^2^ = 5.87, *p* > 0.05), nor for children (χ^2^ = 4.93, *p* > 0.05) and also no significant interaction of PrimeType with Vincentiles (adults: χ^2^ = 15.31; children: χ^2^ = 6.54, both *p* > 0.05). The relevant contrasts did not reach significance either.

## Discussion

The present study sought to examine the underlying mechanisms of morphological processing of word and nonword primes in German adults and children beyond mean response times by extending the analysis to response time distributions. Besides replicating previous results for masked morphological priming in German-speaking adults, the aim was to explore whether priming in the nonword paradigm affects the whole RT distribution (shift) or only parts of it (skew) and whether this is different in the different priming conditions, indicating different mechanisms. Secondly, we were interested in how the results for adults pertain to masked priming in elementary school children.

Results for adults showed robust priming effects for suffixed words (*kleidchen-KLEID*) and also suffixed nonwords (*kleidtum-KLEID*) relative to both nonsuffixed nonwords (*kleidekt-KLEID*) and unrelated words (*träumerei-KLEID*). This pattern replicates earlier findings (Rastle et al., 2004; Longtin and Meunier, 2005; McCormick et al., 2009) showing that adults automatically decompose morphologically complex letter strings into stem and suffix independently of semantics and regardless of the lexical status, which can be interpreted as morpho-orthographic segmentation (for related evidence for derived nonwords in a non-priming task in German, see Bölte et al., 2009a,b). Additionally, the significant facilitation from nonsuffixed nonwords (*kleidekt-KLEID*) relative to unrelated words (*träumerei-KLEID*) is in line with recent findings using morphologically complex nonword primes (Morris et al., 2011; Beyersmann et al., 2015a, 2016) and indicates some amount of embedded stem priming in the absence of an affix, albeit this is significantly smaller than in the presence of an affix. This adds to the growing evidence in favor of an embedded stem priming mechanism in addition to morpho-orthographic segmentation by affix-stripping. Taking into account the RT distribution by use of quantiles, we observed a shift, not a skew, of the RT distribution in the two suffixed conditions as well as in the non-suffixed condition, relative to the unrelated condition. This can be best interpreted in terms of an immediate pre-activation, providing a headstart for target processing. This headstart mechanisms that has also been observed by Andrews and Lo (2013) for transparent, opaque and form-related word pairs thus pertains to the processing of nonword primes.

The results for adults obtained in the present study are in line with morphological processing accounts that suppose early sublexical decomposition, such as obligatory decomposition accounts (e.g., Taft, 2003), form-then-meaning accounts (e.g., Rastle and Davis, 2008) or hybrid models (e.g., Diependaele et al., 2009). While obligatory decomposition and form-then-meaning accounts propose that all complex words must undergo an initial morpho-orthographic segmentation, hybrid models assume that morpho-orthographic decomposition can occur in parallel with whole-word processing of complex words. In all three accounts, successful morpho-orthographic decomposition of the prime would pre-activate the target, manifesting in a shift of the RT distribution. However, strict form-then-meaning accounts (e.g., Rastle and Davis, 2008), which posit a rigid chronological order of semantically blind (morpho-orthographic) and later semantically informed (morpho-semantic) decomposition, fit our results less well. These accounts would predict differences between priming from suffixed words and suffixed nonwords both with regard to magnitude of priming and pattern of the RT distributions, which we did not find. Moreover, our results speak against supralexical accounts (e.g., Giraudo and Grainger, 2001), which presume that morphological decomposition happens after whole-word activation and then sends activation to morpheme representations. Under those accounts, priming from suffixed nonwords is not plausible and a skew rather than a shift of the RT distribution would have been expected due to feedback activation. Amorphous theories (Baayen et al., 2011) that regard morphological effects as the convergence of form and meaning cannot be fully ruled out by our study. However, we consider them less likely due to the finding that suffixed word and nonword primes yielded equal priming in our study, which amorphous models do not account for. Taken together, our results speak in favor of hybrid accounts or obligatory segmentation that is not solely driven by affix-stripping, adding to the growing evidence on stems as salient activation units in morphological processing (Morris et al., 2011; Beyersmann et al., 2015a, 2016).

Turning to the results for children, developing readers also showed significant facilitation from real suffixed words compared to unrelated words. In addition, faster response times were observed following suffixed and nonsuffixed nonwords relative to unrelated words. Importantly, in contrast to adults, the difference between the suffixed and nonsuffixed prime conditions did not reach significance in developing readers, which suggests that there was no evidence for morpho-orthographic decomposition by means of affix-stripping in these individuals. Presumably, elementary school children instead activate embedded stems through partially shared orthography, as Beyersmann et al. (2015b) reported for proficient child readers. This is consistent with the pattern observed in the quantiles, suggesting that there was a shift rather than a skew in the RT distribution of the suffixed word, suffixed nonword, and non-suffixed nonword conditions. Although the shift pattern was less consistent for children than for adults, it speaks in favor of an early embedded stem activation mechanism in German elementary school children.

With reference to morphological processing accounts, again hybrid models seem to best explain the priming pattern of both mean RTs and RT distributions of the children in our study, because these models incorporate a whole-word processing route that allows for embedded stem priming. Embedded stems are mapped onto orthographic whole-word representations, even if the overlap is only partial (see also Ziegler et al., 2014). Embedded stems might thus function as lexical representations that can be activated automatically during the early stages of visual word recognition (Beyersmann et al., 2015b). In a transparent language like German, where an alphabetic reading strategy is usually accurate and efficient, elementary school children could still be prone to read sequentially from left to right. Consequently, this would allow for the activation of words embedded at the beginning of the input letter string, independently of what follows (be it suffix or nonsuffix). An interesting test of this assumption would be an analogous masked priming study with prefixed primes that feature the stem in the second position instead of the first position. Another closely related possibility for the interpretation of our results is that children already use some prestage of morpho-orthographic decomposition, in which abstract affix representations are not yet sufficiently fine-tuned to allow the reliable segmentation into stem and affix (see also Castles et al., 2007). Hence, developing readers would decompose every item that features a stem and a relatively frequent ending. Proper morpho-orthographic segmentation would only be established later on in reading development, arguably through repeated co-activation of stems and their derived forms (see also Rastle and Davis, 2008; Grainger and Ziegler, 2011; Beyersmann et al., 2012). The later acquired morpho-orthographic representations of affixes, would then be used to decompose any stimulus that *appears* to be morphologically complex (whether it is a truly suffixed word, a pseudosuffixed word or a suffixed nonword), but not stimuli that feature nonsuffix endings. It thus appears that the activation of embedded stems via the whole-word route represents an important prerequisite for the later acquisition of more fine-tuned morpho-orthographic representations throughout reading development. Unfortunately, open questions remain about the nature of the embedded stem priming process in children, in particular whether they happen at a lexical or orthographic level.

Future studies would need to address specifically whether the embedded stem priming effect observed in children should be attributed to higher-order lexical processes or lower-level orthographic processes. This would not only be beneficial for models of morphological processing, but also for models of reading development. Moreover, the replication of the present pattern using other paradigms—for example go/no-go lexical decision, which is less demanding for children (Moret-Tatay and Perea, 2011)—could be helpful in order to ensure the reliability of the effects from the arguably more difficult and specific yes/no decision task. With regard to the distributional analysis, extending the exploratory non-parametric approach to more advanced parametric analyses follow-up studies would profit from aiming at more advanced parametrical methods like ex-Gaussian or Weibull analyses would allow a more precise picture of the distributions of priming effects. However, for those analyses a larger number of data points per condition is crucial to draw sensible conclusions.

In summary, examining masked morphological priming with nonwords beyond mean response times through taking into account response time distributions yielded interesting new insights into the mechanisms of morphological decomposition. Adults showed equal facilitation with a shift of the response time distribution from both suffixed words and suffixed nonwords, indicating morpho-orthographic decomposition as an early and automatic pre-activation process independent of lexical status. They also showed quantitatively smaller, but qualitatively similar facilitation from nonsuffixed nonwords, indicating additional embedded stem priming. Children showed equal facilitation from real suffixed words, suffixed nonwords, and nonsuffixed nonwords, suggesting that German elementary school children rely on the activation of embedded stems rather than segmentation of morpho-orthographic reading units by affix-stripping. Our findings suggest that distribution analyses present a promising tool to look beyond mean RTs (Yap et al., 2008). One important extension of our work would therefore be the use of parametrical methods for distributional analyses. This promises to provide more precise insights into the time-course of morphological processing mechanisms and especially the role of embedded stems in skilled as well as developing readers.

## Author contributions

All authors substantially contributed to the present paper. The study was designed and planned by JH, EB, and SS. JH performed data collection, JH and SS analyzed the data. All authors discussed the results and implications. JH drafted the manuscript and EB and SS critically revised it. All authors approved the final version to be submitted and agree to be jointly accountable for all aspects of the work.

### Conflict of interest statement

The authors declare that the research was conducted in the absence of any commercial or financial relationships that could be construed as a potential conflict of interest.

## Appendix

**Table A1:** **Experimental primes and word targets used in the experiment (excluding filler items and nonword targets)**.

**Suffixed word prime**	**Suffixed nonword prime**	**Nonsuffixed nonword prime**	**Unrelated word prime**	**Word target**
stückchen	stücklos	stückau	trepplein	STÜCK
kleidchen	kleidtum	kleidekt	träumerei	KLEID
pferdchen	pferdei	pferdekt	spieglein	PFERD
steinchen	steintum	steinpern	wolkenlos	STEIN
spielchen	spiellein	spielnauf	herzogtum	SPIEL
reichtum	reichlein	reichekt	birnlein	REICH
heiligtum	heiliglos	heiligarf	enkelchen	HEILIG
wachstum	wachslein	wachspern	freudlos	WACHSEN
irrtum	irrchen	irrnauf	endlos	IRREN
eigentum	eigenlos	eigenarf	brauerei	EIGEN
tischlein	tischtum	tischnauf	metzgerei	TISCH
sternlein	sternei	sternarf	kaisertum	STERN
herzlein	herztum	herzekt	kraftlos	HERZ
kindlein	kindei	kindpern	teilchen	KIND
hemdlein	hemdei	hemdnauf	trostlos	HEMD
bäckerei	bäckerchen	bäckerau	tantchen	BÄCKER
zauberei	zauberlein	zauberekt	altertum	ZAUBER
fischerei	fischerlos	fischerau	stimmchen	FISCHER
gärtnerei	gärtnerlos	gärtnerarf	brauchtum	GÄRTNER
prügelei	prügelchen	prügelarf	bildchen	PRÜGELN
hilflos	hilfchen	hilfpern	esserei	HILFE
lautlos	lautchen	lautpern	hexerei	LAUT
arbeitslos	arbeitei	arbeitau	menschlein	ARBEIT
sprachlos	sprachlein	sprachau	besitztum	SPRACHE
spurlos	spurtum	spurnauf	rehlein	SPUR
wohnung	wohnheit	wohnucht	fäulnis	WOHNEN
hoffnung	hoffheit	hoffmen	rundlich	HOFFEN
landung	landig	landucht	wirrnis	LANDEN
impfung	impflich	impfucht	torheit	IMPFEN
drehung	drehlich	drehmen	staubig	DREHEN
grünlich	grünig	grünatz	sammlung	GRÜN
merklich	merknis	merkpfen	erlebnis	MERKEN
glücklich	glückig	glückatz	kribbelig	GLÜCK
ärgerlich	ärgerung	ärgeram	schmutzig	ÄRGERN
sportlich	sportung	sportam	gleichnis	SPORT
rutschig	rutschheit	rutschmen	festlich	RUTSCHEN
schuldig	schuldnis	schulducht	wahrheit	SCHULD
dreckig	drecklich	dreckam	hoheit	DRECK
hungrig	hungrung	hungratz	neuheit	HUNGER
frostig	frostnis	frostam	süßlich	FROST
geheimnis	geheimig	geheimatz	erfindung	GEHEIM
finsternis	finsterung	finstermen	gesundheit	FINSTER
hindernis	hinderheit	hinderam	friedlich	HINDERN
wildnis	wildlich	wildpfen	prüfung	WILD
erlaubnis	erlaubheit	erlaubucht	wanderung	ERLAUBEN
schönheit	schönlich	schönpfen	neugierig	SCHÖN
freiheit	freiung	freipfen	bewegung	FREI
dunkelheit	dunkelnis	dunkelmen	vorsichtig	DUNKEL
krankheit	kranknis	krankpfen	elterlich	KRANK
dummheit	dummig	dummatz	kenntnis	DUMM
